# Medical errors and consequent adverse events in critically ill surgical patients in a tertiary care teaching hospital in Delhi

**DOI:** 10.4103/0974-2700.50740

**Published:** 2009

**Authors:** Sunil Kumar, Sujata Chaudhary

**Affiliations:** Department of Surgery, University College of Medical Sciences and Guru Teg Bahadur Hospital, Shahdara, Delhi-110 095, India; 1 Department of Anesthesiology, University College of Medical Sciences and Guru Teg Bahadur Hospital, Shahdara, Delhi-110 095, India

**Keywords:** Adverse events, medical errors, peritonitis, trauma

## Abstract

**Background:**

Medical errors and adverse events (AE), though common worldwide, have never been studied in India. We believe that though common these are under reported. Aim: The aim of this study was to study medical errors and consequent AE in patients presenting with trauma and bowel perforation peritonitis.

**Methods:**

Five hundred and eighty-six consecutive patients with trauma or peritonitis, presenting to surgery emergency of UCMS-GTBH, were prospectively studied using review form (RF) 1 and 2. AE was defined as an outcome not expected to be part of the illness. RF 1 was filled for all and indicated if AE was present or not. RF2 was filled when RF 1 indicated presence of AE; it further confirmed the occurrence of AE and pointed to the type of medical error and resultant disability. All results were expressed as percentage.

**Results:**

There were 500 (85%) males. Mean age of the patients was 31 years. There were 332 patients with peritonitis and 254 with trauma. AE and its consequences were present in 185 (31.5%) and 183 (31.2%) patients, respectively. Consequences were as follows: disability – 157 (85%), increased hospital stay and/or increased visits in the OPD – 28 (15.3%) and both-101 (55.2%) patients. Disabilities were: death – 62 (40%), temporary disability – 90 (58%) and permanent disability – 05 (3.1%) patients. AE in 133 (71.8%) patients was definitely (level of confidence 6) due to error in healthcare management. All AE were considered preventable. Error of omission accounted for AE in 122 (65.9%) patients. System and operative errors were the commonest, 84.3% and 82.7%, respectively. One hundred and sixty-seven (90%) patients had multiple errors.

**Conclusions:**

The study proves that medical errors and AE are a serious problem in our set-up and calls for immediate system improvement.

## INTRODUCTION

Medical errors and consequent adverse events (AE) in the practice of medicine are common. These have been reported to be 8th commonest cause of death from un-natural events in USA.[1] The annual cost of treating medical injuries in USA has been estimated to be $9 billion.[2] Likewise, 850,000 AE occur annually in NHS hospitals, and put additional burden of £2 billion a year.[3] Fortunately, half of these AE are avoidable and 30%-50% complications are preventable by adopting “safe systems.”[4,5] However, in our country, hardly any work has been done on medical errors, AE and development of safe systems. This study has been done to find the magnitude of the preventable AE and to answer other related questions in critically ill surgical patients attending surgery emergency of a tertiary care teaching hospital in Delhi. The ultimate aim is to create awareness towards developing safe systems and improving quality of care – it is a small step towards that long journey.

## MATERIALS AND METHODS

This prospective study was done in surgery unit III (now Surgery unit II) at University College of Medical Sciences and Guru Teg Bahadur Hospital, Delhi, India. This surgical unit is responsible for care of all emergency patients presenting to surgery emergency on every Monday, Thursday and every third Sunday. The hospital's Ethical Committee had approved the study. The enrollment of the study began in January 2002 and finished in December 2005. All patients presenting to surgery emergency on abovementioned days constituted the universe of the patients. From this large pool, patients admitted with a diagnosis of injury or bowel perforation peritonitis (nontraumatic and pathological, e.g., duodenal perforation, typhoid perforation, etc.) were included for the study. These are the two most common types of surgical emergencies that we encounter, making up almost 80–85% of all general surgical emergency admissions. By studying these two conditions, we ensured that large sample size is collected in short time and findings of this study can possibly be applied to the remaining general surgical emergency patients because the study covers 80–85% patients.

All patients were investigated and managed according to their individual needs. A team of resident doctors with one senior resident as the team leader was responsible for the care in emergency area. As a policy in this unit, the resident doctors discuss the complex cases with the consultant on-call for finalizing management. The consultants directly supervised care, investigations and treatment of all enrolled patients as soon as possible after admission and thereafter on day-to-day basis by monitoring the progress. Patients were ordered discharge by the consultants when patients met following criteria: accepting feeds orally, moving bowel, ambulating, afebrile and healed or healing wounds. All AE occurring during hospital stay of the patients were recorded and managed accordingly. The two authors reviewed all records of discharged patients one or two days before the proposed discharge date. The records of dead patients were reviewed within 48 h of the event.

Following records were reviewed for all the patients included in the study: casualty medical officer's notes, surgery resident doctor's notes, operation, anesthesia and postoperative notes if applicable, daily progress notes in the ward, all original investigation reports, staff nurse's records and discharge summary or death certificate (as applicable). Autopsy reports were reviewed in medico-legal cases.

For the purpose of study, review forms (RF) 1 and 2 were obtained from Mr. Charles Vincent, UK. These forms were suitably modified to meet the local needs. The two authors trained the final year postgraduate students for filling RF1. Questions in RF1 decided if criteria for AE and the AE itself were present or not. Questions in RF2 confirmed (or otherwise) presence of AE, decided the level of confidence with which we could say that the AE was due to various issues related to healthcare management, and helped us taking decisions on preventability of error, class of error (error of omission or commission), type of error (e.g., diagnostic, medication, operative, anesthetic etc.) and effect of AE on patient (type of disability- temporary, permanent or death, increased hospital stay or increased OPD visits). Both authors filled RF2 jointly after examination of the patient-related records mentioned above.

Levels of confidence have been defined in box 1.

**Box 1:** Definitions of levels of confidence

**Definition of various confidence levels for causal relation between AEs and errors:**
Virtually no evidence for management causation
Slight to modest evidence for management causation
Management causation not likely; less than 50-50 but close call
Management causation more likely than not, more than 50-50 but close call
Moderate or strong evidence for management causation
Virtually certain evidence for management causation
**Definition of various confidence levels for preventability:**
Virtually no evidence for preventability
Slight to moderate evidence for preventability
Preventability not quite likely; less than 50-50 but close call
Preventability more likely than not; more than 50-50 but close call
Strong evidence for preventability
Virtually certain evidence for preventability

The data was compiled using MS-excel and percentages were calculated.

## RESULTS

A total of 586 patients (peritonitis – 332; trauma – 254) were studied during the study period. There were 500 males (85.3%). The age ranged from 0 to 80 years, with mean (th ±SD) being 31 (±16) years. More than 70% patients (n = 401) were in the age group of 11 to 40 years. Table 1 shows distribution of patients in age-blocks of ten.

**Table 1:** Age distribution of the patients (*n* = 586)

Age (years)	Frequency (%)
0–10	41 (7.0)
11–20	133 (22.7)
21–30	155 (26.5)
31–40	123 (21.0)
41–50	69 (11.8)
51–60	32 (5.5)
>61	33 (5.6)

As per the preliminary assessment using RF1, the criteria for AE and the AE itself were present in 217 (37%) patients. On filling RF2, however, the AE was found to have occurred in 185 (31.5%) patients only. The remaining 32 patients either died or suffered other unrelated events (such as angina, myocardial ischemia, bronchospasm, chest infection, urinary tract infection, retention of urine, etc.) on account of the risk factors inherent to the patients rather than due to healthcare management issues. The AE had some form of impact in 183 (31.2%) patients; various impacts were: disability in 157 (85%), increased hospital stay and/or increased visits in the OPD in 28 (15.3%) and both disability as well as increased stay/OPD visits in 101 (55.2%) patients. The disabilities were further categorized as temporary, permanent and death. Of the total disabilities (157) seen, death occurred in 62 (40%), temporary disability occurred in 90 (58%) and permanent disability occurred in five (3.1%) patients. The over-stay in the hospital ranged from 1 to 60 days with a mean (±SD) of 9.9 (±12.9) days. The AEs in the other two patients (with no impact such as disability, increased hospital stay/OPD visits, or both) were self limiting and minimal pneumothorax while inserting long line via subclavian vein and deposition of about 100-ml dextrose saline in the soft tissues of the forearm used for intravenous access.

As per the level of confidence, AE in 133 (71.8%) patients was definitely (level of confidence 6) due to error in healthcare management. Level of confidence ranged from 2 to 5 in 50 (27%) patients, and it was 1 in two (1.6%) patients. Thus, all (185) AEs were attributable to errors. Similarly, all (185) were considered preventable, although for 11 (6%) AE the level of confidence for deciding preventability was <3.

AE in 122 (65.9%) patients was thought to be due to errors of omission, and in 63 (34.1%) patients, it was believed to be due to error of commission. Table 2 shows the frequency of errors. System (typical examples of which are given in Box 2) and operative errors (either related to wrong choice or wrong step) were the commonest, 84.3% and 82.7%, respectively. Medical procedure-related errors were the least common, just 1.6%.

**Box 2:** Examples of system errors

Defective/nonavailability of healthcare equipments, instruments, disposables, drugs, supplies, etc.
Nonavailability of the protocol/policy/plan (e.g., lack of infection control programs)
Inadequate training or supervision of doctors or other personnel
Delay in provision or scheduling of services (e.g., lab tests, x-rays, follow-up visits)
Inadequate reporting or communication (e.g., resident reporting late or not reporting at all due to failure of communication system)
Inadequate staffing involving various departments (e.g., during shift changes, weekends, holidays)
Inadequate functioning of hospital services (e.g., pharmacy, blood bank, housekeeping, etc.)
Lack of team work
Inappropriate/inadequate discharge plan

**Table 2:** Types of errors in descending frequency (*n* = 185)

Type of errors	Number (%)
System errors	156 (84.3)
Operative errors	153 (82.7)
Diagnostic errors	40 (21.6)
Clinical (postoperative) management related	22 (11.8)
Drug and fluid administration (medication) related	06 (3.2)
Anesthesia related	05 (2.7)
Medical procedure related	03 (1.6)

Table 3 shows the frequency with which multiple errors occurred. There was no patient in whom all seven errors occurred. One hundred and sixty seven (90%) patients had 2–6 errors. Only 18 (10%) patients had single error. Table 4 enlists the frequency with which AE occurred; the commonest were wound-related infections.

**Table 3:** Multiplicity of errors (*n* = 185)

	Number (%)
All (7) errors involved	Nil
5–6 errors involved	06 (3)
3–4 errors involved	34 (18)
2 errors involved	127 (69)
Only 1 error involved	18 (10)

**Table 4:** Complications (*n* = 185)

	Number (%)
Wound-related complications	86 (46.5)
Anastomotic leak	19 (10.3)
Missed perforations/injuries	09 (4.8)
Iatrogenic injuries	04 (2.2)
Others	22 (11.9)

Time taken to complete RF 1 was recorded in all but one patient; it ranged from 5 to 45 minutes with mean (±SD) being 11.3 min (±4.2). Time taken to fill RF 2 was recorded in all patients; it ranged from 1 min to 90 min with the mean (±SD) time being 8.2 (±9.1) min. It took more time to fill RF1 and 2 in the beginning. However, as we gained experience, the time to fill these forms came down drastically without loosing reliability of data collection and analysis.

## DISCUSSION

Inability to achieve the intended aim, either because of failure to complete a planned action or failure to plan, is defined as an error.[6] Undesirable outcome or deviation from the expected course of the disease is defined as adverse event (AE).[7] AE are consequential (to errors).

Medical errors and resultant AE is a global phenomenon. However, lack of uniform method of documentation and investigation does not allow accurate assessment of its true incidence. Nonetheless, its consequences are huge, both in terms of death as well as financial burden.[1–3,8] Furthermore, it is generally agreed that estimates are mostly conservative as underreporting of errors is frequent.[9] Thus, the incidence of errors and AE may be alarmingly high in developing countries, as is proved by this maiden study.

AE may result from errors related to diagnosis, anesthesia, operative procedure, medical procedures, clinical (postoperative) management, drug and fluid (medication) administration and healthcare system. Previously, it was believed that the medication errors were the commonest.[10] However, current estimates put its incidence close to less than 5%.[11,12] In our study, these errors accounted for less than 4% of AE. Computerized physician order entry has proved to be effective in reducing the medication errors,[13] although its exact role in high volume setups such as ours is debatable.

Medical procedure related errors were rare in this study for the obvious reason that such procedures were rarely performed on acutely ill surgical patients. Anesthesia errors were also rare. This may well be because of the lead taken by anesthetists in recognizing inherent safety issues, objective assessment of the patient problems and implementation of many technological innovations like pulse oximeters and capnographs.[14]

In our study, majority of AE were due to errors related to healthcare system, operative procedure and finally diagnosis. High number of system errors underlines the need for such studies. More importantly, it underlines the need for urgent development of safe systems in surgery, just like anesthesia. Optimal safe system for surgical patients has to incorporate a number of aspects that have been clearly defined.[15]

The second most common type of error in our study was operative error. It is alarming to have such a high number of operative procedure-related errors. Operative errors as the cause of most of the AE have been reported earlier too.[16] Most errors in surgical services have been shown to begin in operation theatres.[17] In our institute, most surgical emergencies are managed by the resident doctors, and they are known to make technical errors because of inexperience.[17] Strategies like expanded time-out and crew resource management may help reduce operative errors.[18,19]

The third commonest error in our study was the diagnostic errors. These may be related to one or several aspects of the complex process of patient evaluation (such as poor choices in diagnostic testing or data interpretation, surgeon's cognitive disposition or attitude, “probability of diagnosis” considerations and lack of diagnostic guidelines).[20,21]

Generally speaking, single medical error does not translate itself into an AE.[22] In our study too, multiple errors accounted for as many as 90% AE. Since in this study, we had concentrated on preventable errors, it is important to note that in 94%, the level of confidence for preventability was ≥3. This point needs to be kept in mind while comparing our results with studies showing lesser number of preventable AE.[4]

A wide variety of AE were seen but wound-related complications (such as infection and dehiscence) were the commonest. This may be due to the fact that the large number of patients in our series had intra-abdominal sepsis. AE may result into death, permanent (bodily) disability, temporary (bodily) disability or merely increased hospital stay and or outpatient visits. In our study, all AE had one or the other type of impact on the patient outcome. Financial implications, likely to be enormous, were not computed as these were outside the scope of this paper. This may be a potential area for future work.

Traditionally, AE have been blamed on individuals. However, studies from high risk-industries such as nuclear reactor and aviation make strong case for shifting focus from individuals to system failures for occurrence of AE in medicine, too.[5] Two types of system failures, active and latent, have been recognized.[23] Active failures are the errors occurring at the point of interaction between the care provider and the system. Thus, these are the unsafe acts committed by the healthcare provider(s). On the other hand, latent failures arise in the background of organization and the work culture, training and competence of staff, control and monitoring proesses.[24] These have been summarized as inevitable resident pathogens. The ‘holes' or ‘faults' in the latent conditions g*et al*igned to allow the trajectory of medical error to result in AE.[23] For this reason and in combination with the fact that it is usually not possible to influence the active failures, good defense mechanisms rely on prevention of latent failures. The operation theatres are the commonest seat for inception of AE in the hospitals in surgical patients,[25] and breakdown in team work and communication have been identified as the root cause of errors in elective as well as emergency surgery settings.[26–28]

All industries like medicine and aviation, involving human employees, have inherent, though variable, risk of errors because fatigue, excessive workload, poor communication or assessment, etc. adversely affect the decision making ability of humans working therein, making them prone to commit errors.[29] Further, like pilots, doctors overestimate their ability.[30] But aviation industry has benefited from the use of promotion of culture of safety as critical and integral part of designing safe system. Undoubtedly, we have to adopt similar risk management strategies to deal with medical errors and AE. Three steps that are vital constituent of “safe systems” include (i) steps that prevent errors, (ii) steps that unmask errors in the event of their occurrence and (iii) steps that mitigate the effects of errors, again in the event of their occurrence.[31] Person-approach to error management finds its mention only to be condemned as (i) the roots of most unsafe acts lie deeply buried in the faults in the systems, (ii) it suffers from other serious shortcomings and (iii) it does not allow development of safety culture.[23] Occurrence of a large number of system errors in our study verifies the need to continue to lay stress on system approach.

Surgery is inherently unsafe. This is reflected by large number of operative errors, second only to system errors in our study. In particular, for preventing operative procedure-related errors, standardization of procedures, making checklists, voluntary error-reporting and positive culture changes (like team work) and teamwork training have been advocated.

Interventions necessary to prevent the AE shall be guided primarily by the types of errors one finds. Other guiding factors are more broader and general such as the country, institution, culture and precise audit of other barriers.

There are a few drawbacks in this study. That the study takes into account only the preventable AE is the most important one. However, since it is not an interventional study the exact proportion of the preventability is not known. Further, the study takes into account a wide variety of system errors. Probably, it would have been better to focus on a few important system errors like physical presence of consultant during emergency surgery.

To conclude, this study underlines the urgent need of population-based studies from this sub-continent to find the magnitude of the medical errors and AE. However, our findings are significant enough to conclude that nation wide system changes in our healthcare institutions are long due to prevent medical errors and AE.
